# Are Urban-Canopy Velocity Profiles Exponential?

**DOI:** 10.1007/s10546-017-0258-x

**Published:** 2017-06-08

**Authors:** Ian P. Castro

**Affiliations:** Aerodynamics and Flight Mechanics, Faculty of Engineering and the Environment, University of Southampton, Highfield, Southampton, SO17 1BJ UK

**Keywords:** Canopy flows, Urban environment, Velocity profiles

## Abstract

Using analyses of data from extant direct numerical simulations and large-eddy simulations of boundary-layer and channel flows over and within urban-type canopies, sectional drag forces, Reynolds and dispersive shear stresses are examined for a range of roughness densities. Using the spatially-averaged mean velocity profiles these quantities allow deduction of the canopy mixing length and sectional drag coefficient. It is shown that the common assumptions about the behaviour of these quantities, needed to produce an analytical model for the canopy velocity profile, are usually invalid, in contrast to what is found in typical vegetative (e.g. forest) canopies. The consequence is that an exponential shape of the spatially-averaged mean velocity profile within the canopy cannot normally be expected, as indeed the data demonstrate. Nonetheless, recent canopy models that allow prediction of the roughness length appropriate for the inertial layer’s logarithmic profile above the canopy do not seem to depend crucially on their (invalid) assumption of an exponential profile within the canopy.

## Introduction

Probably the first suggestions that the spatially-averaged axial mean flow profile (*U*(*z*) versus *z*) within roughness canopies is exponential were made by Inoue (1963) and Cionco (1965), in the context of vegetation canopies. There have since been numerous demonstrations from both field and model (laboratory) experiments confirming such behaviour (Finnigan 2000), which arises largely because both the sectional drag coefficient and the mixing length do not vary significantly with height within a canopy of plants (trees or tall crops). The momentum equation then reduces to a particularly simple form that implies exponential behaviour of *U*(*z*) within the canopy. This profile transforms smoothly to the usual above-canopy logarithmic behaviour and there must always be an inflection point in the profile around the top of the canopy. Vegetation canopies have been widely explored (see, for example, Poggi et al. 2004; Böhm et al. 2013, who used model canopies in a laboratory flume and a wind tunnel, respectively). It is well-known that the dynamics of the flow in such canopies are not too dissimilar to those of the plane mixing layer (Raupach et al. 1996), largely because of the inflection point.

Nicholson (1975) and, much later, MacDonald (2000) undertook some (laboratory) experiments on flows through arrays of cubes and suggested that exponential profiles pertain even in that very different type of canopy. It must be noted, however, that they were unable to obtain vertical profiles genuinely averaged across the entire space between obstacles and had to rely on averaging just a few, hopefully representative, profiles. This continues to be a significant problem for experimentalists and even using particle image velocimetry combined with laser Doppler anemometry it is very difficult, if not impossible, to achieve fully three-dimensional spatial averaging *within* the canopy (see Reynolds and Castro 2008, as an example of what *can* be done).

In recent years numerous authors have reported the results of either direct numerical simulation (DNS) or large-eddy simulation (LES) experiments for flows over and within (mostly urban-type) canopies comprising arrays of sharp-edged obstacles. Such computations naturally yield far more information than can be obtained from any conceivable field or laboratory experiment but it should not be forgotten that there is just as much scope for error in producing computational results as there is in the laboratory. Accuracy depends not least on the quality of the mesh, the fidelity of the numerical methodology, the adequacy of the imposed boundary conditions, and (for LES) the appropriateness of the subgrid model. For all computations employed herein, these (and other relevant) issues have been carefully addressed and, where possible, solutions compared with quality laboratory data, as fully discussed in the original literature.

A major advantage of computational solutions is that the results can be used to extract full spatially-averaged data, not just for the mean flow variables but also for turbulence quantities including the dispersive stresses—stresses that arise because of the spatial variability of the time-averaged quantities within the canopy. Kono et al. (2010) used such information from their LES studies of cube canopies of various plan area densities, $\lambda _p$, to explore quantities within the canopies such as the sectional drag coefficient and the mixing length. ($\lambda _p$ is defined as the ratio of the top surface area of the cube and the repeating unit floor area upon which it resides.) It was shown that neither was constant with height, but the consequent lack, or otherwise, of good exponential fits to velocity profiles was not explored. On the other hand, Yang et al. (2016) have recently argued *for* the exponential behaviour of the mean velocity on the basis of their LES studies of cube canopies, but they did not explore either sectional drag or mixing length variations. Coceal and Belcher (2004) were perhaps the first to suggest that mean velocity profiles were *not* generally exponential in such canopies, but this was on the basis of simulations using the Reynolds-averaged Navier–Stokes equations made with the UK Met Office BLASIUS model with a first-order turbulence closure scheme. Nonetheless, their demonstration that a varying (rather than constant) mixing length led to a non-exponential velocity profile is significant. Here, we present crucial canopy flow and drag information from a number of extant computations. Although many of the results of these computations have previously been published, the datasets were not analyzed at the time to generate the information necessary to assess the adequacy of the assumptions used to develop canopy models analytically. It turns out that, as Coceal and Belcher (2004) suggested, mean velocity profiles within the canopy do not generally have an exponential shape, in contrast to vegetation canopies, and the reasons for this are confirmed.

A summary of the datasets mined for the present work is presented in the next section, without detailed explanations of how they were obtained; those can all be found in the original papers, many (but not all) of which originated from the author’s collaborators. Sections 3 and 4 show mean flow profiles and the quantities normally used to develop canopy models, for canopies ranging from what would usually be considered ‘sparse’ (i.e. widely spaced obstacles) to denser ones leading almost to skimming-type flows. Some discussion and conclusions are presented in Sect. 5.

**Fig. 1:**
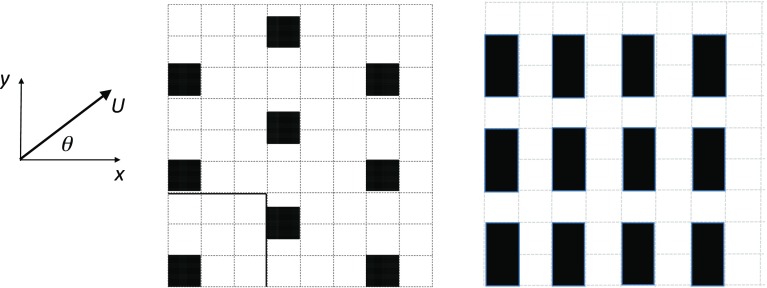
*On left* plan view of a typical cube array—a staggered array with $\lambda _p=0.11$ (Leonardi and Castro 2010). Aligned arrays would, for $\lambda _p=0.25$ for example, have *cubes* in *boxes* 1, 3, 5 $\ldots$ (or 2, 4, 6, $\ldots$ ) along alternate rows in the figure. *At right* plan view of DIPLOS array of $h\times 2h\times h$ cuboids (Castro et al. 2016)

## The Flows Considered

Much of the data presented here has been derived from the DNS studies of Leonardi and Castro (2010). These were half-channel flow simulations in which the wall was covered with staggered arrays of cubes with various area densities, as measured by the usual plan area density. In these and the other cases described below (unless stated otherwise) the flow was aligned normal to obstacle faces. For cubic obstacles $\lambda _p$ (defined above) is identical to the frontal area density, $\lambda _f$—the ratio of the frontal area of the obstacles to the floor area of the repeating unit. The study covered the range $0.04\le \lambda _p\le 0.25$, i.e. from quite sparse to quite dense roughness. The cube height *h* was one eighth of the channel half-height (*H*) and there were typically 12 cubes within the computational domain, independent of $\lambda _p$. The mesh was particularly fine, especially over the cube height, having a grid size of $\varDelta =h/100$. This vertical grid dimension is the most crucial. Some computations introduced below used uniform (square) grids but in others the horizontal grid dimension varied, but was always at its most refined near the obstacle walls; values of $\varDelta$ always refer to the vertical grid dimension within the canopy. A typical roughness array is shown in Fig. 1. The data of Coceal et al. (2006) and Branford et al. (2011) are also considered; they performed DNS on both staggered and aligned (sometimes called ‘square’) arrays of cubes having $\lambda _p=0.25$ in channels with $H=8h$ using DNS but with a somewhat coarser resolution ($\varDelta =h/32$). A similar set of flow data derives from the recent study of Cheng and Porte-Agel (2015) (see also Cheng and Porte-Agel 2016), who used LES to compute a spatially-developing boundary-layer flow, in a domain of height $H=12.6h$. The uniform mesh had a grid size of $\varDelta =h/16$, and an areal density range of $0.028\le \lambda _p\le 0.25$ was considered. Two wind-direction cases were studied ($\theta =0^{\circ }$ and $27^{\circ }$, Fig. 1). LES, for the staggered array with $\lambda _p=0.25$ but over a range of wind directions and in a half-channel with $H=4h$, have also been reported (Claus et al. 2012); grid sizes were typically $\varDelta =h/25$ over the canopy height. A ‘random height’ extension of the case of the staggered cube array with $\lambda _p=0.25$ was that initially studied experimentally by Cheng and Castro (2002) and subsequently computationally by Xie et al. (2008), using LES for a channel flow with domain height $H=10h$. This canopy had block heights of $0.28h_m$, $0.64h_m$, $h_m$, $1.36h_m$ and $1.72h_m$, with the numbers of blocks of each height adjusted to yield a Gaussian height distribution (with mean height $h_m$) within the entire domain. The mesh had a resolution of about $\varDelta =h_m/16$ in the canopy region. More recently, LES of boundary-layer flow over staggered and aligned cube roughness arrays with $\lambda _p=0.03$–0.25 has been reported by Yang et al. (2016). They used a boundary-layer height $\approx 24h$ but a resolution of only $\varDelta =h/8$. For the present work, data from the two sets of boundary-layer simulations (Cheng and Porte-Agel 2015; Yang et al. 2016) were spatially averaged only within the fully developed region downstream. A final set of data is that reported by Castro et al. (2016) who, as part of a wider dispersion project (DIPLOS—http://www.diplos.org), used a channel-flow LES for a square array of rectangular obstacles of size $2h\times 1h\times 1h$ spaced apart by 1*h* (Fig. 1) and at three flow angles, $\theta =0^{\circ }$, $45^{\circ }$, and $90^{\circ }$, in domains with $H=12h$. These three cases all have the same $\lambda _p$ (0.33) but varying $\lambda _f$ of 0.33, 0.35 and 0.17, respectively, for the three flow angles. Table 1 summarises all of these various computations.

**Table 1:** Details of the various datasets used

Case	Method	Acronym: Authors	*H* / *h*	$\varDelta {/}h$
Staggered cubes	DNS	LC: Leonardi and Castro (2010)	8.0	1/100
Staggered and aligned cubes	DNS	CTCB: Coceal et al. (2006)	8.0	1/32
Staggered and aligned cubes	DNS	BCTB: Branford et al. (2011)	8.0	1/32
Staggered cubes, various angles	LES	CCTBBC: Claus et al. (2012)	4.0	1/25
Staggered, random height	LES	XCC: Xie et al. (2008)	10.0	1/16
Aligned cubes (b.layer)	LES	CP-A: Cheng and Porte-Agel (2015)	$\approx 12$	1/16
Staggered and aligned cubes (b.layer)	LES	YSMM: Yang et al. (2016)	$\approx 24$	1/8
Aligned $h\times 2h\times h$ blocks	DNS, LES	CXFRCHHC: Castro et al. (2016)	12	1/12

Except for CP-A’s and YSMM’s LES, where *H* refers to the approximate boundary-layer depth, all cases were channel-flow computations, with *H* the channel half-height. All cases except CCTBBC and CXFRCHHC considered only arrays that were flow-aligned—i.e. wind direction normal to the faces of the obstacles. For the LES cases, the subgrid models used were either the standard Smagorinsky model (CCTBBC, XCC, CXFRCHHC), the modulated gradient model of Lu and Porte-Agel (2010) (CP-A), or the Vreman (2010) model (YSSM)

In discussing the canopy region results presented below, possible differences arising from different domain sizes or outer flow conditions (e.g. whether of boundary-layer or channel type) are generally assumed to be small, in line with Castro et al. (2016). We address the influences of Reynolds number, resolution and (in LES cases) subgrid model where appropriate. In all cases Reynolds numbers based on canopy height and wall friction velocity ($u_\tau$, determined by the imposed axial pressure gradient in the channel cases) was $\mathcal {O}(1000)$ so that each flow was in the fully rough regime.

## Mean Flow Profiles

**Fig. 2:**
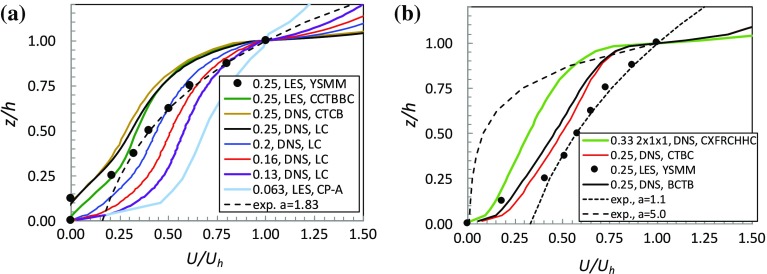
Canopy spatially-averaged mean velocity profiles for cases of flow-aligned arrays. Values of $\lambda _p$ are given in the legends. **a** Staggered array; **b** aligned array. The legends indicate data sources: YSMM (*black circles*), Yang et al. (2016); CCTBBC (*green*), Claus et al. (2012); CTCB (*brown*), Coceal et al. (2006); LC (*black, blue, red, purple*), Leonardi and Castro (2010); CXFRCHHC (*green*), Castro et al. (2016); BCTB (*black*), Branford et al. (2011); CP-A (*green*), Cheng and Porte-Agel (2016)

All field variables shown are spatial and time averages. Within the canopy these are *extrinsic* spatial averages—i.e. averages over the total volume, rather than the fluid volume only, which would yield *intrinsic* averages. Böhm et al. (2013) outline the differences between these two averaging methods and the topic has recently been explored more fully by Xie and Fuka (2017, in press). Figure 2 shows an initial selection of spatially-averaged, axial mean velocity profiles within the canopy derived from some of the datasets identified in Sect. 2. The velocities have been normalized by $U_h$, the velocity at $z'=z/h=1$, the arrays are either of staggered (Fig. 2a) or aligned (Fig. 2b) type, and the LES data from the recent computations of Yang et al. (2016) are shown using symbols. Also plotted in Fig. 2a is the exponential profile defined by $U/U_h=\text {exp}[a(z'-1)]$, where *a* is a constant. Yang et al. (2016) found that a value of $a=1.83$ worked well for their LES data. Note, firstly, that these latter data (in Fig. 2a) do not collapse onto the other three sets for exactly the same roughness geometry ($\lambda _p=0.25$), whereas these other three agree between themselves reasonably well, at least above $z/h=0.5$, with only small differences in the bottom half of the canopy in the LES data (Claus et al. 2012). The Yang et al. (2016) computations used a coarse grid over the cube height ($\varDelta =h/8$) and it is perhaps unsurprising that this is insufficient to capture the relatively sharp shear layer seen at the canopy top in all the other computational profiles.

It is reasonable to assume that the DNS data (with a resolution in *z* of $\varDelta =h/100$) are the most accurate. Note, secondly, that these data (and the somewhat less resolved but very close DNS data of Coceal et al. 2006) for $\lambda _p=0.25$ yield small negative velocities near the surface. This is because the reversed flow regions behind each cube in this case are not so small that spatially-averaged velocities at low heights become positive, although they clearly do for less dense canopies (smaller $\lambda _p$). The finest resolution LES data for $\lambda =0.25$ (Claus et al. 2012) differ marginally from the corresponding DNS data below $z/h=0.5$, perhaps either because of imperfections in the subgrid model or inadequate grid resolution or a combination of both. However, it is more likely an effect of the factor of two difference in domain height. The Claus et al. runs used a height of only 4*h*, which is probably small enough to generate partial suppression of the full extent of the reversed flow regions behind the cubes.

**Fig. 3:**
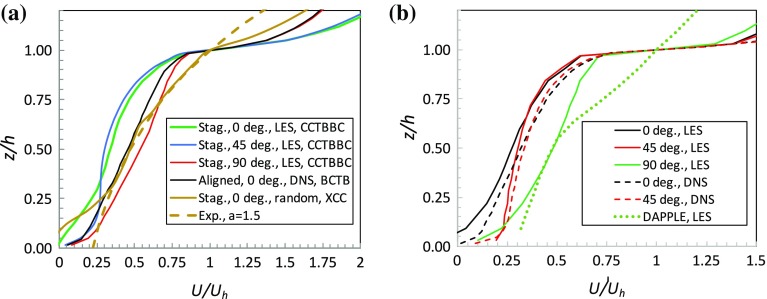
Canopy spatially-averaged mean velocity profiles for $\lambda _p=0.25$. **a** Staggered and aligned arrays, various wind directions; CCTBBC (*green*), Claus et al. (2012); BCTB (*black*), Branford et al. (2011). The *dashed line* is exponential with $a=1.5$, fitting the profile for the case of staggered blocks of random height, Xie et al. (2008) (XCC, *brown*), which is plotted using $h=h_m$ and $U/U_h=U/U_{h_m}$. **b** The DIPLOS array at various wind angles, from Castro et al. (2016), and the DAPPLE array, from Xie and Castro (2009) ($\lambda _p=0.53$), with a flow angle of about $51^{\circ }$ to the major street direction. This case was *not* modelled as a channel flow and thus required appropriate turbulent boundary-layer inlet conditions, as explained by Xie and Castro (2009)

Thirdly, it is clear that the sharp interface at the canopy top weakens with a reduction in $\lambda _p$ (Fig. 2a), not surprisingly, and is also present for the aligned arrays (Fig. 2b) in which the blocks are directly behind one another in the successive rows. These results are similar to those shown by Kono et al. (2010). No value of *a* yields a reasonable exponential fit, simultaneously capturing this interface and the rather slower decay in *U* below it. Figure 2b includes an exponential profile with $a=5$ as a somewhat extreme example, which manages the former rather better, but not the latter. As in the staggered case, the Yang et al. (2016) data and corresponding exponential (with $a=1.1$) differ significantly from the better-resolved DNS data. It would seem that if a sufficiently coarse grid is used the sharp shear layer interface between the canopy flow and the flow aloft is not captured and exponential velocity profiles may result.

Figure 3 shows similar profiles to those shown in Fig. 2 but emphasizing the effect of flow orientation rather than $\lambda _p$. In the upper half of the canopy, staggered arrays of cubes at wind angles of $\theta =0^{\circ }$ and $\theta =45^{\circ }$ yield similar profiles (Fig. 3a). On the other hand, once $\theta =90^{\circ }$, canopy velocities are significantly higher, no doubt because for this direction (unlike the other two) there are uninterrupted streets in the flow direction. Likewise, for the array of $h\times 2h\times h$ obstacles and $\theta =90^{\circ }$ (Fig. 3b) the long uninterrupted streets have only short openings between their ends, whereas for $\theta =0^{\circ }$, (the orientation shown in Fig. 1) the street intersections are of the same size as the regions between obstacles in the same row. An aligned array of cubes, similarly, yields rather higher canopy velocities because of the uninterrupted streets. Note, incidentally, that the drag of the staggered array is higher than that of the aligned array and highest for $\theta =45^{\circ }$, because $\lambda _f$ is highest for that case and there are no uninterrupted streets of any width in the flow direction (Castro et al. 2016).

Figure 3b also includes data from LES of a region in the centre of London, from Xie and Castro (2009) who modelled the field and wind-tunnel case extensively explored in the DAPPLE project (Dobre et al. 2005). This has numerous obstacles of various shapes and sizes and, unlike all the other situations considered here, is thus a much more general type of urban canopy. The value of *h* used for this case is the height of the largest building (about $1.45\times$ the mean building height). In only one of the cases shown in Fig. 3 is there any possibility of an exponential fit over any substantial height range (i.e. exceeding, say, 0.1*h*) within the canopy but this one exception is of some interest. It is the case of staggered blocks of random heights studied using LES by Xie et al. (2008). The velocity profile in Fig. 3a clearly shows a much less sharp shear layer around the top of the canopy or, rather, around its mean height $h_m$, than all the other (uniform height) canopies. This is a natural expectation and is fully discussed in Xie et al. (2008). Perhaps because of this thicker shear layer with its inevitably smaller velocity gradient, an exponential profile (with $a=1.5$) does provide a reasonable fit in the range $0.25<z/h_m<1$, despite the slight ‘bumps’ that can be seen in the profile at the different heights of the various blocks. But in the upper half of the canopy ($1<z/h_m<1.72$) the exponential fit fails and if, alternatively, the full canopy height ($1.72h_m$) and the velocity at that height are used to normalize *z* and *U*(*z*), respectively, no exponential fit is possible (not shown). The same is true for the DAPPLE case included in Fig. 3b (Xie and Castro 2009), which has variable building heights and shapes in the array and is discussed later.

We consider the implications of these two random height cases in due course but turn now to consider the behaviour of the sectional drag coefficients and mixing lengths within the canopy.

## Sectional Drag and Mixing Length Profiles

The time- and space-averaged axial momentum equation for (fully developed) flow at height *z* within a canopy region can be expressed as

1 $$\begin{aligned} \left( -\frac{1}{\rho }\frac{\partial P}{\partial x}\right) -\frac{\partial \overline{uw}}{\partial {z}}-\frac{\partial \tilde{u}\tilde{w}}{\partial z}+\nu \frac{\partial ^2 U}{\partial z^2}=D(z) \end{aligned}$$

where $-\overline{uw}$ is the usual time- and space-averaged Reynolds shear stress, $-\tilde{u}\tilde{w}$ is the dispersive stress associated with momentum transport by the spatial deviations of the ensemble mean velocity field from the spatially-averaged mean axial velocities and $\nu$ is the kinematic viscosity. The bracketed term usually only appears in cases of channel-flow computations and is the axial pressure gradient that provides the total driving force used to generate the flow and so equals $u_\tau ^2/H$. (The friction velocity is defined by $u_\tau =\sqrt{\tau /\rho }$ with $\tau$ the surface stress and, typically, the appropriate force is applied within each cell of the domain.) For reasonable domain heights ($H>>h$) this additional term is small compared to the other terms within the canopy, and it is henceforth ignored. Incidentally, in cases of open channel flow over rough beds, such as rivers for example, the bracketed term is replaced by the gravitational driving force dependent on the bed slope. There is a considerable literature on this general topic; e.g., see Nikora et al. (2004), for typical references and discussion about possible velocity profiles.

*D*(*z*) in Eq. 1 is the canopy drag force, a drag per unit volume of air produced by the combination of pressure differences across the canopy obstacles and viscous forces on their side surfaces. In all the developments of canopy models in the literature the dispersive stress and viscous contributions are ignored. At the Reynolds numbers used for the computations explored herein the viscous stresses can be ignored but, as will be shown later, the dispersive stresses are generally not negligible. The importance or otherwise of dispersive stresses within canopies has been explored previously; an example is Poggi et al. (2004) who, in the context of vegetative-like canopies, showed that they are only significant for sparse canopies. (Incidentally, in summarizing the earlier literature, they also stated that Cheng and Castro (2002) found negligible dispersive stresses in urban-type canopies. This is incorrect as these authors did not measure dispersive stresses within the canopies.) Likewise, very recently and again in contrast to all the present cases, Boudreault et al. (2017) have shown that for forest canopies the dispersive stresses are only important near the edge of the forest and (in some cases) very near the top of the canopy.

**Fig. 4:**
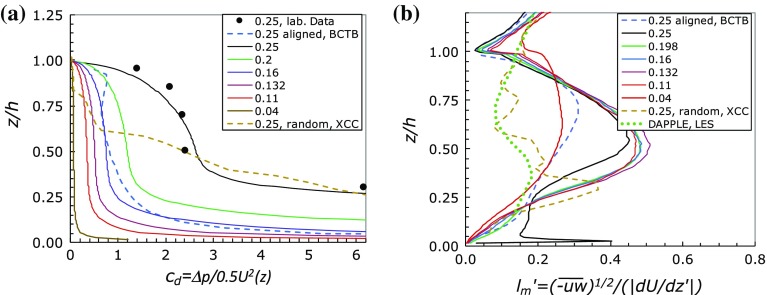
**a** Sectional drag coefficient within the canopy. All data for a staggered array of cubes with the $\lambda _p$ values given in the legend, from Leonardi and Castro (2010), except for the aligned array data from Branford et al. (2011) (BCTB, *blue dashed*) and the random height array of Xie et al. (2008) (XCC, *brown dashed*). The *solid circles* are experimental values obtaining during the course of the laboratory study of Cheng and Castro (2002). **b** Canopy mixing length, normalized (like *z*) by *h*. Legend as for **a**. Results from DAPPLE (Xie and Castro 2009) are included (*green dashed*)

A sectional drag coefficient $c_d$ can be defined by

2 $$\begin{aligned} D(z)=\frac{1}{2h}c_d(z)\lambda _f|U(z)|U(z). \end{aligned}$$

Making the four assumptions that, (i) $c_d(z)$ is constant with *z*, (ii) the (spatially-averaged) Reynolds shear stress can be modelled by the classical mixing length relation $-\overline{uw}=(l_m(z)dU/dz)^2$, (iii) the mixing length $l_m$ is also constant with *z*, and (iv) the dispersive shear stress and all viscous contributions can be ignored, Eq. 1 reduces to the second and last terms only, a balance between the shear-stress gradient and the obstacle drag force. Then (and only then) can it be easily solved explicitly to yield an exponential behaviour of the canopy velocity *U*(*z*).

The viscous forces can be accurately computed from DNS data, but at the Reynolds number of these computations they can be ignored (though they are not entirely negligible, being typically a few percent of the total drag, as discussed by Leonardi and Castro 2010). The drag coefficient is then simply $c_d(z)=\varDelta p(z)/[\frac{1}{2}\rho U^2(z)]$, where $\varDelta p(z)$ is the axial pressure difference across each array obstacle (averaged across its span). The mixing length $l_m$ can also be deduced from the data and Fig. 4 shows both $c_d$ and $l_m$ for the cases computed by Leonardi and Castro (2010). Including viscous drag contributions at each *z* does not materially change the profiles shown in Fig. 4a. Note, firstly, that only for the less dense arrays ($\lambda _p<0.15$, say) can $c_d$ (Fig. 4a) be considered constant over any non-negligible region of the canopy height. Even in these cases, there remain strong variations below $z/h<0.2$, because the velocities become very small there (see Fig. 3). Conversely, near the top of the canopy, $c_d$ tends to zero at $z/h=1$, since the axial pressure difference must be continuous through the canopy top and it is essentially zero just above $z=h$. Incidentally, it is of interest that the laboratory data for $c_d$ obtained by Cheng and Castro (2002) for $\lambda _f=0.25$ are fairly consistent with the DNS data even at low *z*, despite the uncertainties arising from both limited resolution in the pressure measurements and *U*(*z*) values obtained as an average of vertical profiles at just three locations in the array. Coceal and Belcher (2004) viewed the value of $c_d$ at the lowest height (in Fig. 4a) as spurious and took the data as implying a roughly constant $c_d$ below about $z/h=0.75$, but this is clearly an oversimplification. Note too that $c_d(z)$ in the array of random height blocks computed by Xie et al. (2008) is also far from constant. It is worth emphasizing that the fact that $c_d$ is always relatively small near the top of the canopy does not imply that the actual drag force is small there. Indeed, it is known that the total drag of the array is dominated by contributions near the top of the array (Xie et al. 2008).

In contrast to the staggered arrays, data for the aligned array with $\lambda _p=0.25$ (Branford et al. 2011) yield $c_d$ arguably more constant than the corresponding staggered array and, in fact, Kono et al. (2010) have shown that for such an array $c_d$ is ‘almost constant with height above $z/h=0.1$ with $\lambda _p\le 0.25$’. However, the mixing length profiles for such (aligned) arrays were far from constant and, in fact, very similar in form to those for staggered arrays. Figure 4b shows the $l_m$ data and, again, includes those for an aligned array and the random height array. In no case could the mixing length profile be considered as remotely constant. In every case, even for quite sparse arrays (e.g. $\lambda _p=0.11$), the mixing length at the canopy top is very much smaller than it is around the mid-canopy height, because of the relatively large mean velocity gradient there. In this respect the profiles differ significantly from the model suggested by Coceal and Belcher (2004) as an improvement on the $l_m$ = constant assumption, but they are similar to those determined subsequently (e.g. Coceal et al. 2006; Kono et al. 2010; Cheng and Porte-Agel 2015). Note that the variations in block heights within the random array cause the mixing length profile to be much less smooth than it is for uniform height arrays.

**Fig. 5:**
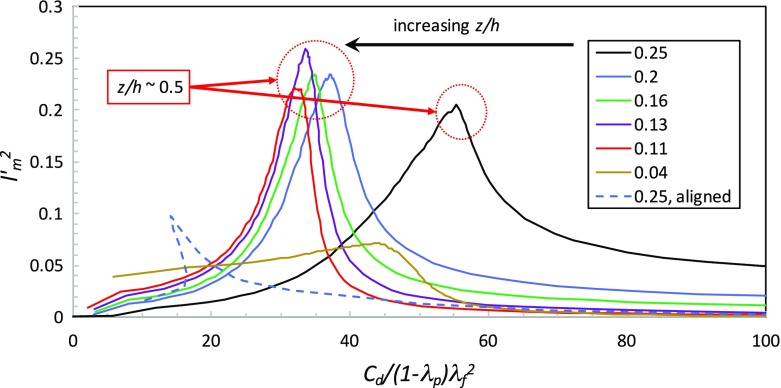
$l_m^2$ versus $c_d/\lambda _f^2$ for the LC data, with $\lambda _p$ values given in the *right window*. Data for the aligned array (BCTB, *dashed lines*) are included. Note the direction of increasing *z* and that the peak mixing length often occurs around mid-canopy height (as evident in Fig. 4b) (Recall that for cube arrays, $\lambda _p=\lambda _f$.)

Accepting the second assumption listed above (i.e. a mixing length relation for the Reynolds shear stress) it is of interest to explore the relationship between $c_d(z)$ and $l_m(z)$ that is then implied by Eq. 2 if the velocity profile is in fact exponential. Using $U=U_h{\hbox {exp}[a(z'-1)]}$ it is straightforward to show that Eq. 1 reduces to

3 $$\begin{aligned} l_m'^2=\frac{\lambda _f}{(1-\lambda _p)4a^3}c_d. \end{aligned}$$

where $l_m'=l_m/h$. Authors who have considered an exponential velocity profile have found that typically $a\sim \lambda _f$ (e.g. Coceal and Belcher 2004; Yang et al. 2016) so Eq. 3 reduces to $l_m'^2\sim c_d/[(1-\lambda _p)\lambda _f^2]$. Figure 5 shows convincingly that this relation does not in fact hold over any height range within the canopy. Note particularly that the aligned-array data ($\lambda _p=0.25$) also do not follow Eq. 3, even though the velocity profile is more closely exponential. Similarly, the random-height-array data (not shown) do not follow $l_m'^2\sim c_d$ or anything close to it.

**Fig. 6:**
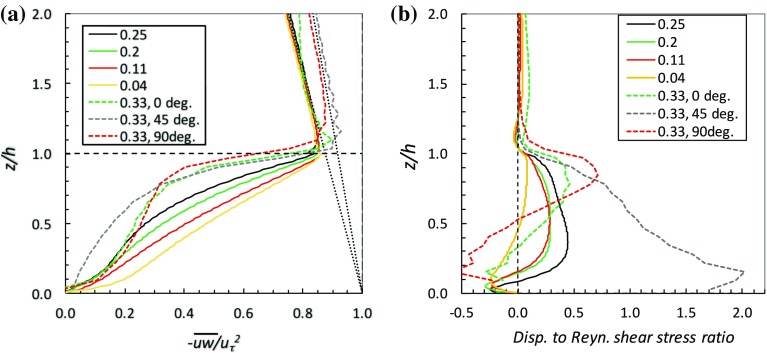
**a** Reynolds shear stress ($-\overline{uw}$) in the canopy. The legend gives values of $\lambda _p$, the *horizontal dashed line* denotes the canopy top. The *dotted lines* are the expected normalised total stress—i.e. all stress components including the pressure contribution across the obstacles—(running from (1,0) to (0,8) for the domain with height 8*h* and (1,0) to (0,12) for the domain with height $12\,h$). Data are from Leonardi and Castro (2010) and (for the $\lambda _p=0.33$ cases, *dashed lines*) Castro et al. (2016). **b** The ratio of dispersive ($-{\tilde{u}\tilde{w}}$) to Reynolds ($-\overline{uw}$) shear stresses

In addition to the failure of the standard $c_d$ and $l_m$ assumptions, it turns out that the dispersive stresses are far from negligible. Figure 6 shows the variation of the Reynolds shear stress with height and the ratio of the dispersive shear stress to that Reynolds stress for selected cases from Leonardi and Castro (2010) and Castro et al. (2016). In all cases the Reynolds stresses fall from their peak values around the top of the canopy towards zero at the bottom, as expected. It is of interest that the stress ratio (Fig. 6b) can be negative, i.e. the dispersive stress can be of opposite sign to the Reynolds stress. This may largely be a result of coherent vortices within the canopy (Rasheed and Robinson 2013) since the sign of the dispersive stress depends on the signs of the axial and vertical mean ‘dispersive’ velocities; i.e. differences between the local *U* and its spatial average and the same for *W*.

**Fig. 7:**
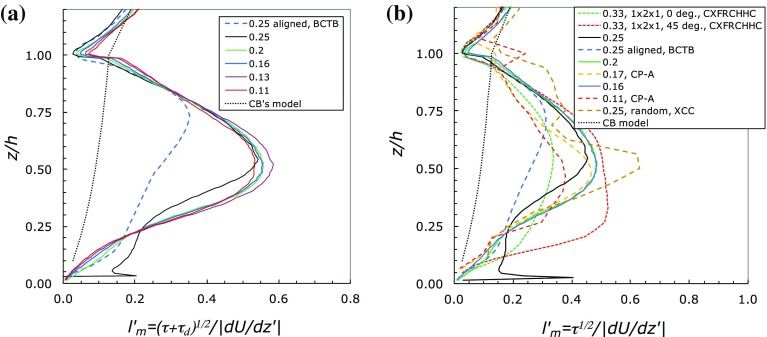
Mixing length profiles. Data from Leonardi and Castro (2010) cases (*solid lines*). In **a**, *dashed blue line* is aligned array data from Branford et al. (2011) (BCTB) and *dotted line* is the Coceal et al. (2006) model (CB); **b** includes data from Cheng and Porte-Agel (2015) (CP-A, staggered cubes in a boundary layer, *long-dashed yellow* and *red*), Branford et al. (2011) (BCTB, aligned array, *long-dashed blue*), Castro et al. (2016) (CXFRCHHC, aligned $1\,h\times 2\,h \times 1\,h$ blocks, *short-dashed green* and *red*) and random height array, Xie et al. (2008) (XCC, *long-dashed brown*). Figures in legends give $\lambda _p$ values

More importantly, the dispersive stress is rarely small compared with the Reynolds stress. Only for the very sparse array ($\lambda _p=0.04$) could the dispersive stress be considered negligible. The immediate implication is that the dispersive stress term in Eq. 1 should not really be ignored in any attempt to develop a model for the expected velocity profile, at least for $\lambda _p\ge 0.1$. It is therefore of interest to compute the mixing length using the *total* shear stress, $\tau +\tau _d=-\overline{uw}-\tilde{u}\tilde{w}$. Figure 7a shows the results for the Leonardi and Castro (2010) cases; the profiles can be compared with those in Fig. 4b and it is clear that adding in the dispersive stress does not materially alter the shape of the latter profiles, although it typically increases the maximum mixing length values by some 20%.

The mixing length results discussed above are representative of all the data we examined. Figure 7b includes $l_m'$ (computed using the Reynolds shear stress) for a range of other cases for which there are sufficient data. This figure shows that, not surprisingly perhaps, changes in array morphology and orientation lead to significant differences in the mixing length profiles. Although in every case the general pattern is very similar (cf. Fig. 4b), maximum values and the height at which they occur are quite dependent on morphology and wind direction. Note that since including the dispersive stress in $l_m'$ does not significantly change the mixing length profiles the non-linear behaviour of $l_m'^2$ versus $c_d$ shown in Fig. 5 is not greatly affected by inclusion of the dispersive stresses. Modelling the total stress using a mixing length is, in any case, physically rather questionable, even if arguments for modelling $\overline{uw}$ that way might not be too unreasonable (as suggested by Coceal and Belcher 2004).

Figure 7 includes the model of Coceal and Belcher (2004), which can be expressed as

4 $$\begin{aligned} \frac{1}{l_m}=\frac{1}{\kappa z}+\frac{1}{\alpha \kappa (h-d)}-\frac{1}{\kappa h}, \end{aligned}$$

where the last two terms together represent $1/l_c$, a mixing length supposed (for dense canopies) to be constant within the canopy and controlled by the thickness of the shear layer ($h-d$) at the top of the canopy. The length $l_m$ was then modelled as the harmonic mean of this mixing length (chosen to match that in the boundary layer above) and the mixing length near the ground ($\kappa z$), yielding Eq. 4. Coceal and Belcher (2004) chose $\alpha =1$ and then used (4), along with an empirical expression relating *d* to $\lambda _p$ as part of the turbulence closure to the full momentum equation (with $c_d$ taken as constant with *z*). For homogeneous canopies, the resulting velocity profiles were only ‘approximately exponential in the upper part of the canopy, but sparse canopies take on a more logarithmic shape’. They could only compare their data with those obtained in a laboratory experiment discussed by MacDonald (2000); he suggested that the velocity profiles were exponential, but his experimentally determined *U*(*z*) profiles were far from being true spatially-averaged profiles, as Kono et al. (2010) have demonstrated. Although Coceal and Belcher (2004) assumed $c_d$ to be constant with height, as did other authors, they concluded that their more sophisticated mixing length model (not constant with height) ‘had the effect that vertical profiles of spatially-averaged mean velocity are not exponential in urban canopies’. This was an important conclusion, which is substantiated by the present work. The fact that usually $c_d$ is also not constant with height (as with $l_m$) only strengthens this conclusion. But the data also indicate that the Coceal and Belcher (2004) model is deficient in some important respects and cannot generally be expected to be adequate for arbitrary canopy morphologies and wind directions.

## Further Discussion and Conclusions

The fact that coarsely gridded LES for uniform height canopies (e.g. the Yang et al. 2016, data shown in Fig. 2) or canopies that embody buildings of various heights can lead to exponential mean velocity profiles within the canopy (at least, in the latter case, over part of the canopy height) is of interest. Surface morphologies typical of real urban areas almost invariably do not have buildings all of the same height and thus the shear layer around the canopy top is inevitably thicker, with significantly lower velocity gradients (see Xie et al. 2008, for a discussion of this point). One might deduce that this leads more naturally to exponential profiles. However, it should be borne in mind that even in cases of non-uniform height canopies, sectional drag and mixing length are no more constant with height than they are for uniform height cases, as shown above. An example of a real-life situation is provided by the wind-tunnel experiments and the (later) computations undertaken as part of the DAPPLE project (http://www.DAPPLE.org.uk, Dobre et al. 2005), which studied an area of central London surrounding the Marylebone Road. LES computations of flow and dispersion over this area have been reported by Xie and Castro (2009). From the LES results it is possible to compute the spatially-averaged velocity profile within a canopy whose dimensions are 400 m $\times$ 400 m in plan. Within this domain there are 35 (mostly sharp-edged) buildings of various heights ranging from 13.5 to 32 m, with an average height of $h=22\hbox { m}$ and giving $\lambda _p=0.53$. The LES mesh had smallest grid sizes of around *h* / 22 and the computational domain was 10*h* in height. Figure 3b includes the mean velocity profile and it is, indeed, characterized by much smaller velocity gradients than all the others considered here. But it could *not* be fitted by an exponential. The data needed to compute the canopy drag coefficient or the dispersive stresses are not available, but the mixing length results are included in Fig. 4b and it is evident that $l_m'$ is not constant with height.

A clear feature of all the well-resolved simulations discussed herein is that neither the sectional drag coefficient nor the mixing length is anything like constant in any of the canopies comprising sharp-edged squat obstacles. Dispersive stresses are also not insignificant. These facts all lead to mean velocity profiles that do not have exponential features over any significant height range. This is a rather negative conclusion, but it provides a useful warning that analytical models of Eq. 1 using the common but, as it turns out, incorrect assumptions may not necessarily be very helpful, at least in predicting even spatially-averaged flow profiles within canopy regions. The complexities of the inhomogeneous, fully three-dimensional and highly turbulent urban canopy flows are very dependent on the canopy morphology (and wind direction) and make generalizations on spatially-averaged quantities rather problematic, at least insofar as they might be used to develop useful canopy models.

Despite this general conclusion, it is emphasized that for computations of the flow above the canopy, even quite simple urban canopy models can lead to useful results: for example, yielding apparently quite reasonable values of roughness length for the above-canopy logarithmic layer, as Coceal and Belcher (2004) have shown. This is fortunate for, as the present work implies, it would not seem feasible to produce a more sophisticated (differential) analytical urban canopy model for use, for example, in current numerical weather prediction models, which does not make invalid assumptions about the flow within the canopy.

**Fig. 8:**
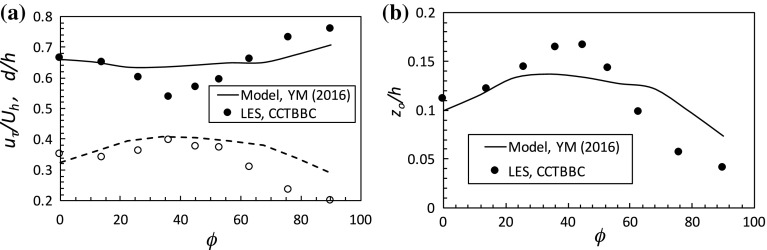
Variations of $u_\tau /U_h$ and *d* / *h* (**a**) and $z_o/h$ (**b**) with wind direction. The *lines* are from the model of Yang et al. (2016) and the *symbols* are from the LES of Claus et al. (2012), both for a staggered cube array roughness with $\lambda _f=0.25$

Rather than starting with the (differential form of the) momentum equation to deduce the velocity profile within the canopy, an alternative approach to the general problem of estimating friction velocity and roughness length is to propose, ab initio, a specific shape function for the velocity profile and use it to develop an algebraic model. Yang et al. (2016) have recently used this approach, with (i) a two-part shape function for the velocity profile (exponential within the canopy and the classical logarithmic law plus wake for the boundary layer above), (ii) the Jackson (1981) definition of the displacement height, and (iii) a geometric wake-sheltering model to provide the unknown parameter (*a*) in the exponential part of the velocity shape function. With appropriate matching conditions at the top of the canopy and with the assumption that the sectional drag coefficient is constant and equal to unity, this led to predictions of $u_\tau$ and $z_o$ as functions of $\lambda _f$, the frontal area density. The model can be employed for arbitrary wind directions (Yang and Meneveau 2016) and Fig. 8 presents the results for a staggered cube array, compared with the corresponding LES results of Claus et al. (2012). This is a rather more complete set of comparisons that those in Yang and Meneveau (2016). (Separate comparisons of $u_\tau$ and $U_h$ normalized by, say, a ‘freestream’ value would not be sensible, since the computations were for a channel flow whereas the model used a boundary-layer profile above the canopy.) Despite the facts that the velocity profile shape within the canopy is not exponential (as the LES results demonstrate, see, e.g., Fig. 3a) and the sectional drag coefficient is not constant, the qualitative features of the model results are not unreasonable, as Yang and Meneveau (2016) concluded. However, quantitatively, the variations with wind direction of all three parameters are significantly smaller than suggested by the LES. Although an alternative velocity profile shape within the canopy could be used in the model, it is less easy to see how the constant sectional drag coefficient assumption could be relaxed and, furthermore, how non-uniform height arrays could be accommodated.

We conclude that, although it is possible to construct both differential and algebraic canopy models that yield fair estimates of (say) the roughness length of the surface for specified, uniform height, rectangular-sectioned obstacle arrays, they each contain some rather limiting assumptions and could not easily be applied to the more varied surface morphologies typical of urban or city centres. There are, nonetheless, recent morphometric models that appear to perform reasonably well, in terms of predicting *d* and $z_o$, even for arrays of variable height buildings. An example is that of Millward-Hopkins et al. (2011), who used an exponential velocity profile in the canopy and a constant sectional drag coefficient, along with a geometric sheltering model not too dissimilar to that of Yang et al. (2016), to estimate *d* and $z_o$, obtaining reasonable agreement with available (but very limited) laboratory data. Most recently, Millward-Hopkins et al. (2013) showed that in cases of variable height buildings, the variability is in fact the most important morphological parameter characterizing the surface. Since height variability is the most common situation in the field, this would seem to be an important conclusion. Whether appropriate relaxations of the (incorrect) velocity profile and sectional drag coefficient assumptions would, even if possible, improve the predictions sufficiently to warrant the extra complexity remains an open question. In any case, there needs to be a wider range of quality datasets (whether from the laboratory or the field or, better still, from LES) against which to validate such models.

It should be emphasized, finally, that the data we have explored all apply to urban-like canopies, i.e. relatively squat, sharp-edged obstacles with various areal densities. The lack of uniformity in sectional drag and mixing length and the consequent lack of an exponential spatially-averaged velocity profile within the canopy is in distinct contrast to the situation for vegetative canopies such as forests or crops that usually embody much more slender, closely packed elements. One might therefore anticipate that slenderness ratio, defined for example as obstacle height divided by cross-wind width (*h* / *w*), would be a significant parameter deter-mining whether a canopy is a more urban or a vegetative type. There has been some effort to address this (see, for example, Huq et al. 2007, who showed that mean flow profiles can be quite different for canopies of buildings with $h/w=3$, compared with canopies of cubes). Very recently, Sadique et al. (2016) have used LES specifically to explore the effect of *h* / *w* by varying it from unity to seven for various $\lambda _p$. They showed that the phenomenological (wake-sheltering) model of Yang et al. (2016), mentioned above and suitably extended to cope with tall obstacles, succeeds quite well in predicting $z_o$ for the above-canopy flow, but they did not explore the canopy region in detail. There is also evidence (Böhm et al. 2013) that largely vegetative-like canopies having more urban-like features appear to be characterized by flows that are only a small perturbation of the classical vegetative canopy flows, in that the turbulence dynamics remain dominated by the mixing-layer type instabilities arising because of the inflection point in the canopy-top velocity profile. These issues remain to be more fully explored.
